# Association between Lipid Levels and Risk for Different Types of Aneurysms: A Mendelian Randomization Study

**DOI:** 10.3390/jpm11111171

**Published:** 2021-11-10

**Authors:** Yanghui Chen, Man Huang, Yunling Xuan, Ke Li, Xin Xu, Linlin Wang, Yang Sun, Lei Xiao, Ping Xu, Wei Kong, Dao Wen Wang

**Affiliations:** 1 Division of Cardiology, Department of Internal Medicine, Tongji Hospital, Tongji Medical College, Huazhong University of Science and Technology, Wuhan 430000, China; chenyh_c@163.com (Y.C.); hm1127697727@163.com (M.H.); xylzzu@163.com (Y.X.); nikosrhee@163.com (K.L.); mambaout8@163.com (X.X.); 13283931569@163.com (L.W.); sunyang.7@163.com (Y.S.); xiaoleihust@163.com (L.X.); 2 Hubei Key Laboratory of Genetics and Molecular Mechanism of Cardiologic Disorders, Huazhong University of Science and Technology, Wuhan 430000, China; 3 Beijing Proteome Research Center, State Key Laboratory of Proteomics, National Center for Protein Sciences (Beijing), Beijing Institute of Lifeomics, Beijing 100000, China; xupingghy@gmail.com; 4 Department of Physiology and Pathophysiology, School of Basic Medical Sciences, Peking University, Beijing 100000, China; kongw@bjmu.edu.cn

**Keywords:** aneurysm, lipid traits, lipid-lowering drugs, mendelian randomization analyses

## Abstract

Background: Although the associations between serum lipid levels and aneurysms have been investigated in epidemiological studies, causality remains unknown. Thus, this study aimed to investigate the causal relationships of serum high-density lipoprotein cholesterol (HDL-C), low-density lipoprotein cholesterol (LDL-C), total cholesterol (TC), and triglyceride (TG) levels on five types of aneurysms, using genetic variants associated with four lipid traits as instrumental variables in a Mendelian randomization (MR) analysis. Methods: We performed two-sample Mendelian randomization (MR) analyses to evaluate the associations of HDL-C, LDL-C, TC, and TG levels with risks for five types of aneurysms and those of LDL-C- (*HMGCR*, *NPC1L1*, *PCSK9*, *CETP*, and *LDLR*) and TG-lowering targets (*ANGPTL3* and *LPL*) with aneurysms. Results: The sample sizes of the included studies ranged from nearly 80,000 to 410,000. We found inverse associations between genetically predicted HDL-C levels and aortic (OR = 0.74, 95% CI = 0.65–0.85) and abdominal aortic aneurysms (0.58, 0.45–0.75). A 1-SD increase in LDL-C and TC levels was associated with increased risks for aortic (1.41, 1.26–1.58 and 1.36, 1.18–1.56, respectively) and abdominal aortic aneurysms (1.82, 1.48–2.22 and 1.55, 1.25–1.93, respectively). TG levels were significantly associated with aortic (1.36, 1.18–1.56) and lower extremity artery aneurysms (2.76, 1.48–5.14), but limited to cerebral aneurysm (1.23, 1.06–1.42). Secondary analyses revealed a relationship between genetically proxied LDL-C-lowering targets and all types of aneurysms; however, the drug targets remained heterogeneous. We found a weak association between TG-lowering therapies and aortic (*ANGPTL3*, 0.51, 0.29–0.89) and abdominal aortic aneurysms (*LPL*, 0.64, 0.44–0.94). Conclusion: According to genetic evidence, lipid dysfunction is a causal risk factor for aneurysms. Lipid-lowering drugs may be a potential effective strategy in preventing and managing aneurysms.

## 1. Introduction

Aneurysms, particularly cerebral and aortic aneurysms, are relatively rare but life-threatening conditions associated with high mortality and morbidity rates. Although the relationship between serum lipid levels and risks for cardiocerebrovascular diseases, such as coronary heart disease, myocardial infarction, and stroke [1,2,3,4], has been well established, the relationship between serum lipid levels and aneurysms remains unclear.

Different types of aneurysms share common risk factors and pathophysiological processes such as vascular remodeling, extracellular matrix degradation, and cell apoptosis. However, dysfunction of lipid metabolism across aneurysms is heterogeneous. In abdominal aortic aneurysm, several genetic studies revealed strong associations of serum high-density lipoprotein cholesterol (HDL-C) and low-density lipoprotein cholesterol (LDL-C) levels with risks for abdominal aortic aneurysms [5,6], and this trend was coincident with the observational studies [7]. However, in cerebral aneurysms, the role of lipid traits, particularly HDL-C, remains controversial. Elevated HDL-C levels contribute to a high risk of aneurysmal subarachnoid hemorrhage and ruptured cerebral aneurysms [8,9]; however, a large cohort of 6411 intracranial aneurysms suggested that higher HDL-C levels are inversely associated with rupture status [10]. Additionally, the current literature lacks studies regarding the role of lipid biomarkers in iliac artery aneurysm, and lower extremity artery aneurysm. In our study, not only abdominal aortic aneurysm but also other four different types of aneurysms were evaluated in terms of the association between lipid-associated single-nucleotide polymorphisms (SNPs) and risks. Additionally, we investigated the association between SNPs in lipid drug targets and risk of five types of aneurysms, which hopes to provide a potential effective treatment strategy for preventing and managing aneurysm.

Mendelian randomization (MR) is a popular approach in genetic epidemiology that circumvents the limitations of observational cohort studies [11]. In MR, genetic variants are usually used as instrumental variables to investigate causal associations, and MR has been widely used to explore the potential effect of exposure on disease [12,13,14]. Additionally, such MR analyses have been performed to determine whether licensed drugs or drug targets are causative [15,16].

Therefore, in this study, we mainly investigated the causal relationship of serum HDL-C, LDL-C, total cholesterol (TC), and triglyceride (TG) levels on five types of aneurysms using genetic variants associated with four lipid traits as instrumental variables in an MR analysis. Furthermore, we assessed the association of LDL-C-lowering targets, including 3-hydroxy-3-methylglutaryl coenzyme A reductase (*HMGCR*), Niemann-pick C1-like 1 (*NPC1L1*), proprotein convertase subtilisin/kexin type 9 (*PCSK9*), cholesteryl ester transfer protein (*CETP*), and low-density lipoprotein receptor (*LDLR)* and TG-lowering targets of angiopoietin-like 3 (*ANGPTL3*) and lipoprotein lipase (*LPL*) with aneurysms.

## 2. Methods

### 2.1. Ethics Statement

Our study was a secondary analysis of the publicly available data. Informed consent was sought for all participants per the original GWAS protocols, and all ethical approvals for the GWAS were obtained by the original GWAS authors.

### 2.2. Study Design

A two-sample MR analysis was conducted to examine the causal relationships of HDL-C, LDL-C, TG, and TC on the risks for various aneurysms, including cerebral aneurysm, aortic aneurysm, abdominal aortic aneurysm, iliac artery aneurysm, and lower extremity artery aneurysm. Electronic health records (ICD-9 or ICD-10 diagnosis and hospital procedure codes) from hospital episode statistics and summary statistics from publicly available genome-wide association studies (GWASs) were used. Secondary analyses were performed to determine the association of genetically proxied inhibition of lipid drug targets with aneurysm risks. The sample sizes of the included studies ranged from approximately 80,000 to 410,000, and all participants were of European ancestry. Participant characteristics and study details are shown in Table S1.

### 2.3. Instrument Identification

Summary data of lipid traits were obtained from a GWAS meta-analysis in the Global Lipids Genetics Consortium, which investigated HDL-C, LDL-C, TG, and TC levels of approximately 190,000 individuals [17]. For each lipid factor, the following inclusion criteria were defined as genetic instrument: (1) significant SNPs with exposure at *p* < 5 × 10^−8^, (2) low-linkage disequilibrium (LD) (defined as *r*^2^ < 0.001), (3) *F*-statistic > 10, (4) significant SNPs with the outcome at *p* > 5 × 10^−8^, and (5) validated by sensitivity analyses based on the MR pleiotropy residual sum and outlier (MR-PRESSO).

In the analyses of drug targets, all polymorphisms within a ±100 kb window on target genes including *HMGCR*, *NPC1L1*, *PCSK9*, *CETP*, and *LDLR*, which are associated with LDL-C, and target genes including *ANGPTL3* and *LPL*, which are associated with TG (*p* < 5 × 10^−8^), were identified. We conducted a clumping procedure to select independent SNPs at an LD *r*^2^ threshold of 0.2 and a physical distance threshold of 250 kb [15].

### 2.4. Summary-Level Genetic Data on Aneurysms

The UK Biobank is a very large and detailed prospective study with over 500,000 participants aged 40–69 years recruited in 2006–2010. All participants provided informed consent to participate in the study and were required to complete a series of baseline measurements. GWAS summary statistics for cerebral aneurysm (N = 408,164), aortic aneurysm (N = 410,793), abdominal aortic aneurysm (N = 409,983), aneurysm of iliac artery (N = 408,718), and aneurysm of artery of lower extremity (N = 408,811) were available in the pan-UKB (https://pan.ukbb.broadinstitute.org.2020, accessed on 19 March 2021) [18]. We also used data on aortic aneurysm (N = 218,972) from the FinnGen R5 release. Additionally, Bakker’s group performed a cross-ancestry, genome-wide association study in 10,754 cases and 306,882 controls of European and East Asian ancestry to discover new risk loci and the genetic architecture of cerebral aneurysms [19]. In our study, we downloaded the dataset from the stage 1 GWAS summary statistics comprising participants (N = 79,429) with European ancestry as an independent replication, as well.

### 2.5. Two-Sample MR Analyses

All MR analyses were conducted using R version 3.6.1 packages “TwoSampleMR” [20] and “MR-PRESSO” [21]. Effect estimates for single SNP were calculated using the Wald ratio method, i.e., by dividing the SNPs outcome association by the SNPs exposure association. The random-effects inverse variance weighted method, which provides a concise estimate and considers potential heterogeneity among estimates from individual variants, was performed to examine the association between multiple SNPs and outcomes [22]. For sensitivity analysis, we applied the following approaches: (1) MR-Egger regression method [23], which provided valid MR estimates in the presence of horizontal pleiotropy when the pleiotropic effects of the genetic variants were independent from the genetic associations with the exposure; (2) weighted median method [24], which provided valid MR estimates under the presence of horizontal pleiotropy when up to half of the included instruments were invalid; (3) weighted mode models [25], which were consistent when the largest number of similar single-variant MR estimates were derived from valid instruments. Additionally, we used *p* < 0.05/4 exposures/7 outcomes = 0.0018 to determine statistical significance. 

As for multivariable MR analysis, a total of 211 SNPs were used in the analysis, which included SNPs which were genome-wide significant in one of HDL-C, LDL-C, and TG. The multivariable IVW method was utilized to assess the blood lipids’ associations on aneurysms with the adjustment of other lipids.

To examine potential directional pleiotropy, we performed MR-Egger intercept analysis to test whether there was evidence of the intercept parameter being different from zero [23]. We also performed the global test, outlier test, and distortion test using MR-PRESSO to detect outliers, which could bias the overall MR estimate [21]. For the analysis of cerebral and aortic aneurysm, statistical heterogeneity was assessed using Cochran Q chi-squared test (significant heterogeneity at *p* < 0.1 and *I*^2^ > 50%). To compare trials exhibiting heterogeneity, pooled data were meta-analyzed using a random-effects model; otherwise, a fixed-effects model was used.

## 3. Results

### 3.1. Genetic Instruments for Lipid Traits

We obtained genetic instruments for four exposures (numbers of instruments: HDL-C = 88, LDL-C = 79, TC = 86, TG = 55) for MR analyses after excluding variants that were in LD (*r*^2^ > 0.01) and in proximity (10 Mb) to other candidate instruments with stronger *p*-values (Table S2). The variance in each exposure explained by instrumental variables ranged from 5.6% to 9.6%. All SNPs had *F* values > 10, suggesting that they were unlikely to introduce marked weak instrument bias in the MR analyses.

### 3.2. Causal Estimates for Lipid Traits on Aneurysm Risks

**Figure 1:**
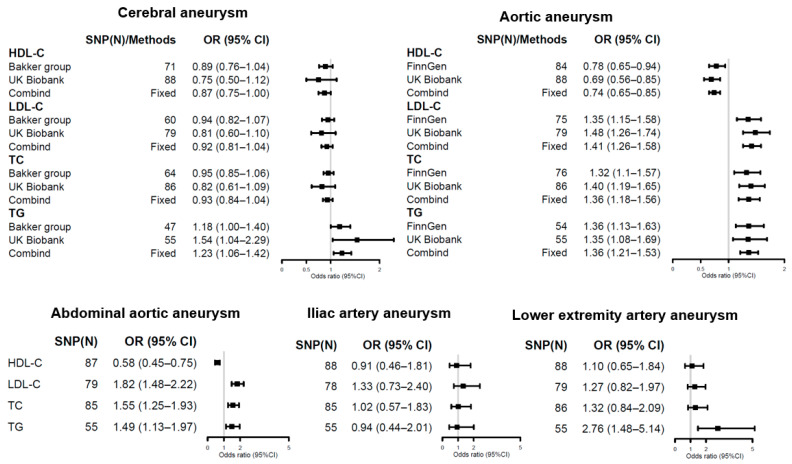
Forest plots showing inverse variance weighted method estimates (odds ratio with 95% CI per 1 SD increase in lipid fraction) of HDL-C, LDL-C, TC, and TG single nucleotide polymorphisms on cerebral aneurysm, aortic aneurysm, abdominal aortic aneurysm, iliac artery aneurysm, and lower extremity artery aneurysm, respectively.

The results of the MR analyses investigating the associations between genetically predicted LDL-C, HDL-C, TC, and TG and aneurysm risk are presented in Table S3 and summarized in Figure 1.

No associations were found between HDL-C (OR = 0.87, 95% CI = 0.75–1, *p* = 0.06), LDL-C (OR = 0.92, 95% CI = 0.81–1.04, *p* = 0.17), and TC (OR = 0.93, 95% CI = 0.84–1.04, *p* = 0.2) levels and cerebral aneurysms. Genetically higher TG levels showed suggestive evidence (0.05 < *p* < 0.0018) for association with cerebral aneurysm (OR = 1.23, 95% CI = 1.06–1.42, *p* = 0.006).

All four lipid exposures were associated with aortic aneurysms after correction for multiple testing. We found significant inverse associations between genetically determined HDL-C levels and aortic aneurysms (OR = 0.74, 95% CI = 0.65–0.85). For 1-SD increase in genetically predicted LDL-C, TC, and TG levels, the combined OR was 1.41 (95% CI = 1.26–1.58), 1.36 (95% CI = 1.18–1.56), and 1.36 (95% CI = 1.21–1.53), respectively, for aortic aneurysms.

Genetically determined HDL-C, LDL-C, and TC levels were statistically associated with abdominal aortic aneurysms. A 1-SD increase in TG levels was nominally associated with a 49% increase in overall abdominal aortic aneurysm risks (OR = 1.49, 95% CI = 1.13–1.97, *p* = 4.8 × 10^−3^); however, this finding was not statistically significant after multiple testing correction.

No significant associations were noted between the four lipid traits and iliac artery aneurysms and lower extremity artery aneurysms, except TG levels. Genetically higher TG levels were significantly associated with an increased risk for lower extremity artery aneurysms (OR = 2.76, 95% CI = 1.48–5.14, *p* = 1.3 × 10^−3^) but not for iliac artery aneurysms (OR = 0.94, 95% CI = 0.44–2.01, *p* = 0.87).

In the multivariable MR analysis, the results showed HDL-C and LDL-C level remained associated with aortic aneurysms (HDL-C, OR = 0.82, 95% CI = 0.73-0.93, *p* = 2 × 10^−3^; LDL-C, OR = 1.14, 95% CI = 1.03–1.26, *p* = 1 × 10^−2^) and abdominal aortic aneurysms (HDL-C, OR = 0.67, 95% CI = 0.43–0.90, *p* = 7.13 × 10^−4^; LDL-C, OR =1.31, 95% CI = 1.10–1.52, *p* = 1.23 × 10^−2^). Additionally, there was also evidence for a direct causal relationship of TG level on risk of cerebral aneurysm (OR = 1.22, 95% CI = 1.02–1.46, *p* = 3 × 10^−2^) and lower extremity artery aneurysms (OR = 2.16, 95% CI = 1.50–2.81, *p* = 2.08 × 10^−2^) (Table S4 and Figure S1).

### 3.3. Genetic Proxies for Lipid Drug Targets and Aneurysm Risk

**Figure 2:**
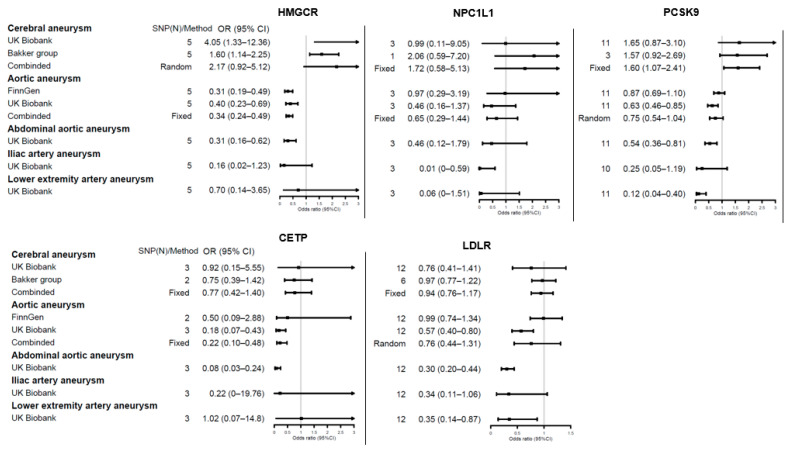
Forest plots showing inverse variance weighted method estimates (odds ratio with 95% CI per 1 SD increase in lipid fraction) of LDL-C-lowering *HMGCR*, *NPC1L1*, *PCSK9*, *CETP*, and *LDLR* single nucleotide polymorphisms on cerebral aneurysm, aortic aneurysm, abdominal aortic aneurysm, iliac artery aneurysm, and lower extremity artery aneurysm, respectively.

**Figure 3:**
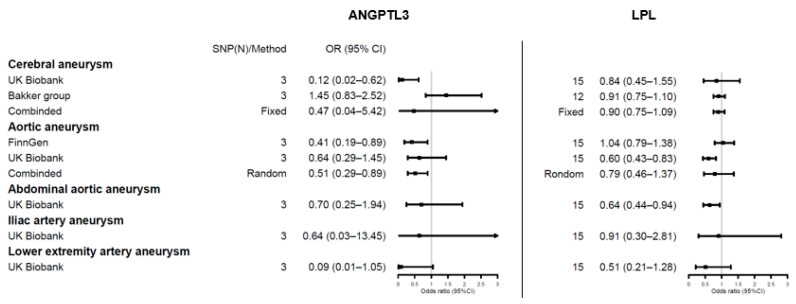
Forest plots showing inverse variance weighted method estimates (odds ratio with 95% CI per 1 SD increase in lipid fraction) of TG-lowering *ANGPTL3* and *LPL* single nucleotide polymorphisms on cerebral aneurysm, aortic aneurysm, abdominal aortic aneurysm, iliac artery aneurysm, and lower extremity artery aneurysm, respectively.

**Figure 4:**
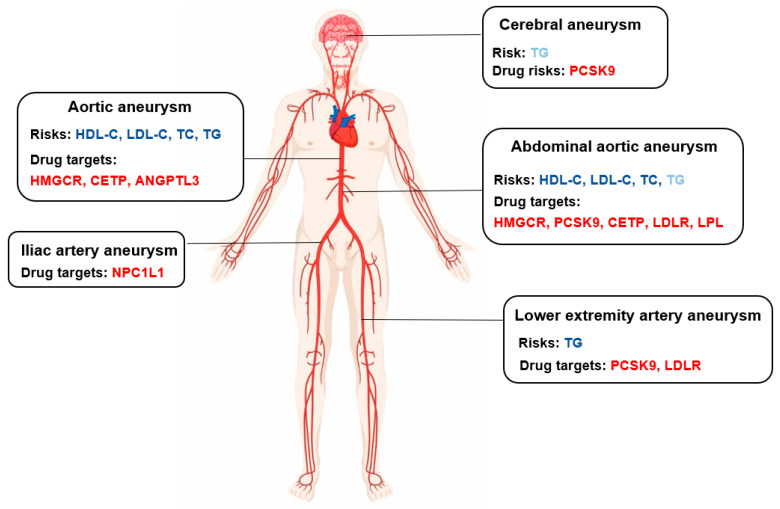
Lipid traits risk and lipid-lowering target in different aneurysms. Blue indicates the potential risk factors in aneurysms, and red indicates the potential protective factors in aneurysms. Dark blue means *p* < 0.0018 for association on lipid levels by inverse variance weighted method and light blue means *p* < 0.05. Red means *p* < 0.05 for association on lipid-lowering target.

The role of lipid levels in aneurysms has been well explored. We thus comprehensively assessed the causal relationships of genetic proxies for anti-lipid drugs on aneurysm risk. Details of the SNP effects are shown in Table S5, and the results are shown in Figure 2, Figure 3 and Figure 4.

The MR analysis revealed an association between genetically proxied LDL-C-lowering inhibition of *HMGCR* with lower risks of aortic aneurysms (OR = 0.34, 95% CI = 0.24–0.49, *p* = 1 × 10^−5^) and abdominal aortic aneurysms (OR = 0.31, 95% CI = 0.16–0.62, *p* = 8.51 × 10^−4^). A consistently strong risk association on cerebral aneurysms in the UK Biobank cohort (OR = 4.05, 95% CI = 1.33–12.36, *p* = 0.014) and Bakker et al. (OR = 1.6, 95% CI = 1.14–2.25, *p* = 6.56 × 10^−3^) was noted, but with moderate heterogeneity between studies (*I*^2^ = 59%, random-effects model, OR = 2.17, 95% CI = 0.92–5.12, *p* = 0.08).

Genetically proxied inhibition of *NPC1L1* was not significantly associated with all types of aneurysms except for a weak association with lower iliac artery aneurysms (OR = 0.01, 95% CI = 0–0.59, *p* = 2.69 × 10^−2^). Protective effects for *PCSK9* were noted with respect to abdominal aortic aneurysms (OR = 0.54, 95% CI = 0.36–0.81, *p* = 2.74 × 10^−3^) and lower extremity artery aneurysms (OR = 0.12, 95% CI = 0.04–0.4, *p* = 5.52 × 10^−4^); however, the meta-analysis revealed a higher risk of cerebral aneurysm with respect to genetically proxied *PCSK9* inhibition (OR = 1.6, 95% CI = 1.07–2.41, *p* = 0.02). We found that genetically proxied *PCSK9* could decrease the risk of aortic aneurysms in the UK Biobank cohort (OR = 0.63, 95% CI = 0.46–0.85, *p* = 3.2 × 10^−3^); however, this was not replicated in the FinnGen cohort (OR = 0.87, 95% CI = 0.69–1.10, *p* = 0.24).

*CETP* variants, resulting in genetically proxied reduction in LDL-C levels, reduced the risks of aortic aneurysms (OR = 0.22, 95% CI = 0.10–0.48, *p* = 2 × 10^−4^) and abdominal aortic aneurysms (OR = 0.08, 95% CI = 0.03–0.24, *p* = 7 × 10^−6^). *LDLR* was associated with a reduction in risk for abdominal aortic aneurysms (OR = 0.30, 95% CI = 0.20–0.44, *p* = 4.54 × 10^−10^) and lower extremity artery aneurysms (OR = 0.35, 95% CI = 0.14–0.87, *p* = 0.024).

Regarding the genetically proxied TG-lowering inhibition, *ANGPTL3* variants were associated with lower risk of aortic aneurysms (OR = 0.51, 95% CI = 0.29–0.89, *p* = 0.02) and cerebral aneurysms in the UK Biobank cohort (OR = 0.12, 95% CI = 0.02–0.62, *p* = 0.012). A similar finding was noted for *LPL* variants on aortic aneurysms in the UK Biobank cohort (OR = 0.60, 95% CI = 0.43–0.83, *p* = 1.95 × 10^−^^3^), but this was not replicated in the FinnGen cohort (OR = 1.04, 95% CI = 0.79–1.38, *p* = 0.78). *LPL* variants were also associated with risks for low abdominal aortic aneurysms (OR = 0.64, 95% CI = 0.44–0.94, *p* = 0.022). 

## 4. Discussion

In this study, we systematically evaluated the causality of the association between four lipid traits (HDL-C, LDL-C, TC, and TG) and aneurysm risks using two-sample MR analyses and found associations between genetically predicted HDL-C, LDL-C, and TC levels and aortic and abdominal aortic aneurysms. TG levels were significantly associated with aortic and lower extremity artery aneurysms, but limited to cerebral aneurysm. 

### 4.1. Controversial Role of Lipid TRAITS in Cerebral Aneurysms

The role of cholesterol on the risk for cerebral aneurysm is unclear. In this study, we did not observe any significant associations of genetically predicted HDL-C, LDL-C, and TC levels with cerebral aneurysms. Similarly, the association between cholesterol-lowering drugs and cerebral aneurysms is controversial. Retrospective studies reported that statin use (*HMGCR* inhibitor) is inversely associated with cerebral aneurysm rupture [10,26]; however, no strong significant beneficial effect or even increased risk of side effects has been noted in clinical trials [27,28].

Previous studies showed PCSK9 inhibitors did not reduce the risk of brain stroke, but newly large clinical studies, such as FOURIER trials, reported its beneficial effects on brain stroke and ischemic brain stroke [29], which might not corresponded to the effect size observed in this analysis. In our study, we found that the inhibition of genetically proxied LDL-C-lowering targets, *PCSK9*, might increase the risk of cerebral aneurysm. This might be because MR estimated the long-term modulation of drug target on the disease risk, and the most relevant limitation of clinical trials is that patients were followed for a relatively short period of time, which might limit definitive conclusions on the long-term effects of extreme cholesterol lowering induced by PCSK9 inhibitors. 

### 4.2. Lipid Dysfunction in Aortic Aneurysms: From Etiology to Drug Therapy

Aortic aneurysms are potentially lethal conditions that cause more than 10,000 deaths per year in the United Kingdom and United States [30,31]. Unfortunately, there is a lack of effective medications for preventing disease progression. Genetic studies have reported some abdominal aortic aneurysm risk loci associated with lipoprotein metabolism [32]. In this study, we provided robust evidence that lipid dysfunction, mainly that of higher HDL-C levels, lower LDL-C, TC, and TG levels, may play a causal role in the etiology of aortic/abdominal aortic aneurysms, and HDL-C and LDL-C level remained associated with aortic aneurysms and abdominal aortic aneurysms after multivariable MR analysis, which was consistent with results of previous observational studies [33,34].

From our analysis, we suggest that lower LDL-C treatment (such as HMGCR inhibitor) might be beneficial to patients. It had been demonstrated that statin use was strongly recommended for patients undergoing vascular surgery, including abdominal aortic aneurysms [35], because statin use could significantly decrease the perioperative morbidity and mortality rates (perioperative strokes, myocardial infarctions, and cardiovascular deaths) [36]. A recent meta-analysis explored the effects of statin use on the outcomes of abdominal aortic aneurysms, and found statin use was associated with significant reductions in long-term and short-term mortality rates [37].

PSCK9 inhibitor is another potential lower LDL-C treatment which was associated with lower odds of abdominal aortic aneurysms from our study. Although there is a lack of clinical trials exploring the effect in aortic aneurysms patients, we believed PCSK9 inhibitor was beneficial to abdominal aortic aneurysm patients. Firstly, PCSK9 plays a critical role in the pathogenesis of vascular diseases. It induces smooth muscle cell dedifferentiation and LDL-C level upregulation via LDLR degradation and upregulates extracellular matrix degradation and adipocytokine expression [38,39,40]. Secondly, the results of recent FOURIER trial showed that adding evolocumab (PCSK9 inhibitor) to statin could reduce the risk of acute arterial events across all vascular territories [29].

Apart from HMGCR and PCSK9 inhibitions, ANGPTL3 might be another potential target for the treatment of aortic aneurysms in our study. To date, targeting ANGPTL3 were emerging in clinical recently, such as evinacumab (a human monoclonal antibody of ANGPTL3) and Antisense oligonucleotides (ASO)-based inhibition of ANGPTL3. These studies showed that ANGPTL3 inhibition resulted in a favorable lipid profile (lower TG and LDL-C levels) and provided potential protection in atherosclerotic cardiovascular diseases [41,42]. There are data to support its beneficial effects on vascular remodeling that could have relevance in aortic aneurysms management [43].

### 4.3. Limited Evidence on Other Aneurysms

Iliac artery aneurysm is a rare vascular disease, but its rupture is associated with high mortality rates. Its main cause is related to progressive atherosclerosis [44]; however, the role of lipid values in the pathogenesis and pathophysiology of iliac artery aneurysms remain unclear. Although we did not observe lipid dysfunction, we speculate that the *NPC1L1* inhibitor likely mitigates the risk of iliac artery aneurysms via an unknown mechanism that might be related to an NPC1L1-specific pathway, which differs from the LDLR pathway.

## 5. Limitations

This study had several limitations. First, although MR had features that minimized the risk for confounding and reverse causation, it was hard to eliminate all the potential influence of directional pleiotropy, causing biased causal effect estimates. In our study, we attempted to control for pleiotropy by using different sensitivity analyses (MR-Egger, weighted median, and weighted mode analyses), but pleiotropic lipid-based pathway still might be represented as the SNPs were connected within the metabolite network and therefore not independent of each other. Second, the Bonferroni correction for multiple tests performed in the sensitivity analyses suggests that some findings may be false positives. Third, our analyses were based on data obtained from the European population, thus limiting the generalizability of our results to other ethnicities. Finally, although we did not observe any association between lipid traits and iliac artery aneurysms, the negative findings might be attributed to a lack of sufficient statistical power to detect an effect.

## 6. Conclusions

In conclusion, we found genetic evidence to support higher LDL-C, TC, and lower HDL-C as a causal risk factor for aortic aneurysm and abdominal aortic aneurysm. Higher TG levels were significantly associated with aortic and lower extremity artery aneurysms, but modestly associated with cerebral aneurysm. In addition to this, interventions that lower LDL-C are likely to be beneficial for all types of aneurysms expect cerebral aneurysm. TG-lowering therapies were associated with lower odds of aortic and abdominal aortic aneurysms.

## Data Availability

The data are available in Supplementary Files and upon request.

## Supplementary Materials

The following are available online at https://www.mdpi.com/article/10.3390/jpm11111171/s1. Figure S1: Forest plots showing multivariable MR estimates (odds ratio with 95% CI per 1 SD increase in lipid fraction) of HDL-C, LDL-C, and TG single nucleotide polymorphisms on cerebral aneurysm, aortic aneurysm, abdominal aortic aneurysm, iliac artery aneurysm and lower extremity artery aneurysm, respectively. Table S1: summary of Genome-Wide Association Studies involved in our study. Table S2: Detailed summary of genetic variants in HDL-C, LDL-C, TC and TG. Table S3: All Mendelian randomization analysis results of lipid traits. Table S4: Multivariable mendelian randomization analysis results of lipid traits with adjustment for HDL-C, LDL-C and TG. Table S5: Detailed summary of LDL-C lowering genetic variants in HMGCR, NPC1L1, CETP, PCSK9, LDLR and TG lowering genetic variants in ANGPTL3, LPL.
