# Supplementary material for: Association between Lipid Levels and Risk for Different Types of Aneurysms: A Mendelian Randomization Study
Source: J Pers Med. 2021 Nov 10;11(11):1171. doi: 10.3390/jpm11111171 (PMC8621501; doi:10.3390/jpm11111171)
Supplement: Supplementary file 1 [file jpm-11-01171-s001.zip › jpm-1338653-SI.pdf]

Title: Association between lipid levels and risk for different types of aneurysms: A Mendelian randomization study

Yanghui Chen[1,2], Man Huang[1,2], Yunling Xuan[1,2], Ke Li[1,2], Xin Xu[1,2], Linlin Wang[1,2], Yang Sun[1,2], Lei Xiao[1,2], Ping Xu[3], Wei Kong[4], Dao Wen Wang[1,2*]

[1]Division of Cardiology, Department of Internal Medicine, Tongji Hospital, Tongji Medical College, Huazhong University of Science and Technology, Wuhan, 430000， PR China

[2]Hubei Key Laboratory of Genetics and Molecular Mechanism of Cardiologic Disorders, Huazhong University of Science and Technology, Wuhan 430000, PR China

[3]State Key Laboratory of Proteomics, Beijing Proteome Research Center, National Center for Protein Sciences (Beijing), Beijing Institute of Lifeomics, Beijing 100000, China

[4]Department of Physiology and Pathophysiology, School of Basic Medical Sciences, Peking University, Beijing, 100000, China

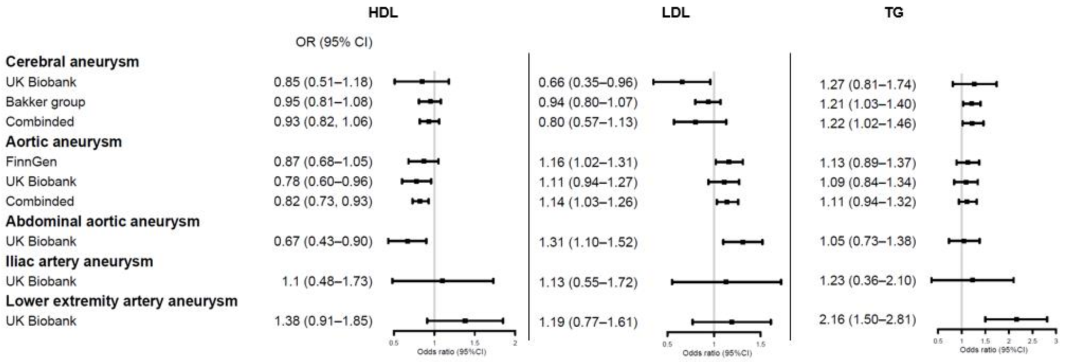

Figure S1. Forest plots showing multivariable MR estimates (odds ratio with 95% CI per 1 SD increase in lipid fraction) of HDL-C, LDL-C, and TG single nucleotide polymorphisms on cerebral aneurysm, aortic aneurysm, abdominal aortic aneurysm, iliac artery aneurysm and lower extremity artery aneurysm, respectively.

Table S1. Summary of Genome-Wide Association Studies involved in our study.

GWAS data set	Cohort	Sample size	Cases	Controls	Ancestry of participants	Pheno sex
HDL-C	the Global Lipids Genetics Consortium	nearly 190,000			European	Both sexes
LDL-C						Both sexes
TC						Both sexes
TG						Both sexes
cerebral aneurysm	UK Biobank	408,164	531	407,633	European	Both sexes
	Mark K. Bakker group	79,429	7,495	71,934		Both sexes
aortic aneurysm	UK Biobank	410,793	2,228	408,565	European	Both sexes
	FinnGen	209,366	2,825	206,541		Both sexes
abdominal aortic aneurysm	UK Biobank	409,983	1,418	408,565	European	Both sexes
iliac artery aneurysm	UK Biobank	408,718	153	408,565	European	Both sexes
lower extremity artery aneurysm	UK Biobank	408,811	246	408,565	European	Both sexes

Table S2. Detailed summary of genetic variants in HDL-C, LDL-C, TC and TG.

Exporsure	SNP	Effect allele	Other allele	Beta	Se	Pvalue	Eaf	F
HDL-C	rs10019888	A	G	0.027	0.0046	4.90E-08	0.8364	34.45179584
HDL-C	rs10087900	G	A	0.0231	0.0036	2.17E-09	0.5607	41.17361111
HDL-C	rs102275	T	C	0.0391	0.0035	6.40E-28	0.628	124.8008163
HDL-C	rs103294	T	C	0.0523	0.0044	4.00E-30	0.186	141.2856405
HDL-C	rs10468017	T	C	0.1179	0.0038	########	0.2757	962.6322715
HDL-C	rs1047891	C	A	0.0269	0.0039	8.73E-10	0.6979	47.57462196
HDL-C	rs10501321	C	T	0.0483	0.0036	3.54E-38	0.314	180.0069444
HDL-C	rs10761771	C	T	0.0198	0.0034	4.12E-09	0.467	33.91349481
HDL-C	rs10808546	T	C	0.0409	0.0034	4.11E-30	0.4459	144.7067474
HDL-C	rs11045163	G	A	0.0217	0.0035	3.20E-09	0.4063	38.44
HDL-C	rs11065987	A	G	0.0222	0.0035	1.23E-09	0.5778	40.23183673
HDL-C	rs11765979	C	A	0.0412	0.0048	3.11E-17	0.4578	73.67361111
HDL-C	rs11789603	T	C	0.06	0.006	3.70E-21	0.08971	100
HDL-C	rs12133576	A	G	0.0243	0.0035	6.15E-11	0.3549	48.20326531
HDL-C	rs12145743	G	T	0.0203	0.0036	1.80E-08	0.3311	31.7970679
HDL-C	rs12412743	C	T	0.0291	0.0045	1.31E-09	0.847	41.81777778
HDL-C	rs12740374	T	G	0.0343	0.0041	1.69E-15	0.2124	69.98750744
HDL-C	rs12748152	C	T	0.0506	0.0062	9.74E-16	0.92876	66.60665973
HDL-C	rs12801636	A	G	0.0235	0.0042	3.15E-08	0.2243	31.30668934
HDL-C	rs13076253	A	C	0.0283	0.0048	4.96E-09	0.8522	34.76085069

HDL-C	rs13099479	A	G	0.036	0.0062	1.82E-08	0.08971	33.71488033
HDL-C	rs13107325	C	T	0.0708	0.0078	1.07E-15	0.92216	82.39053254
HDL-C	rs13702	C	T	0.1058	0.0038	########	0.3127	775.1828255
HDL-C	rs1515110	G	T	0.0323	0.0035	8.04E-18	0.3813	85.16653061
HDL-C	rs1689797	C	A	0.0358	0.0036	2.85E-21	0.6979	98.89197531
HDL-C	rs16942887	A	G	0.0831	0.0051	8.28E-54	0.1332	265.4982699
HDL-C	rs16965220	A	C	0.0219	0.0037	7.91E-09	0.2982	35.03360117
HDL-C	rs17145738	T	C	0.0408	0.0053	4.95E-13	0.1174	59.26094696
HDL-C	rs17173637	T	C	0.0363	0.0057	1.90E-08	0.90237	40.5567867
HDL-C	rs181360	T	G	0.0376	0.0042	9.24E-18	0.8008	80.14512472
HDL-C	rs1866956	T	C	0.0217	0.0037	7.96E-10	0.6755	34.39663988
HDL-C	rs1877031	A	G	0.0336	0.0036	1.20E-19	0.6755	87.11111111
HDL-C	rs1883025	C	T	0.0698	0.0041	1.50E-65	0.7573	289.8298632
HDL-C	rs1936800	C	T	0.02	0.0034	3.06E-10	0.5277	34.60207612
HDL-C	rs1980493	T	C	0.0318	0.0048	3.76E-10	0.8773	43.890625
HDL-C	rs2013208	T	C	0.0254	0.0036	8.92E-12	0.5053	49.7808642
HDL-C	rs205262	A	G	0.0283	0.0039	3.88E-13	0.7335	52.65548981
HDL-C	rs2066714	C	T	0.0453	0.0071	7.26E-10	0.1201	40.70799445
HDL-C	rs2075650	A	G	0.0554	0.0051	9.72E-26	0.8734	117.9992311
HDL-C	rs2241210	G	A	0.0332	0.0035	2.49E-20	0.5528	89.97877551
HDL-C	rs2241770	T	C	0.0989	0.0057	6.78E-60	0.8971	301.0529394
HDL-C	rs2250802	G	A	0.034	0.0038	2.02E-17	0.3193	80.05540166

HDL-C	rs2278236	A	G	0.0331	0.0035	3.19E-18	0.5435	89.43755102
HDL-C	rs2288912	G	C	0.0297	0.0036	7.15E-15	0.4987	68.0625
HDL-C	rs2293889	G	T	0.0312	0.0035	4.27E-17	0.5871	79.4644898
HDL-C	rs2454722	G	A	0.0351	0.0044	3.31E-14	0.1451	63.63688017
HDL-C	rs2602836	A	G	0.0192	0.0034	4.96E-08	0.4274	31.88927336
HDL-C	rs2606736	C	T	0.0246	0.0043	4.80E-08	0.3945	32.72904273
HDL-C	rs2642438	G	A	0.0303	0.0039	7.78E-14	0.7454	60.36094675
HDL-C	rs2925979	C	T	0.0351	0.0037	1.32E-19	0.7045	89.99342586
HDL-C	rs3741414	T	C	0.0296	0.004	6.10E-14	0.1913	54.76
HDL-C	rs3822072	G	A	0.0251	0.0034	4.06E-12	0.5119	54.49913495
HDL-C	rs3861397	A	G	0.024	0.0036	8.40E-11	0.6583	44.44444444
HDL-C	rs4142995	G	T	0.0263	0.0037	9.37E-12	0.6161	50.52520088
HDL-C	rs4148005	T	G	0.0283	0.0036	5.74E-14	0.7005	61.7970679
HDL-C	rs4240624	A	G	0.0818	0.0058	1.32E-45	0.9248	198.9072533
HDL-C	rs424346	T	C	0.0679	0.0113	4.84E-08	0.04881	36.106273
HDL-C	rs4379922	C	T	0.0247	0.0036	9.56E-12	0.3496	47.07484568
HDL-C	rs4465830	A	G	0.0597	0.0044	5.18E-40	0.7982	184.0955579
HDL-C	rs4650994	G	A	0.021	0.0034	6.70E-09	0.5172	38.14878893
HDL-C	rs4660293	A	G	0.0353	0.004	2.86E-18	0.7639	77.880625
HDL-C	rs4846914	A	G	0.0479	0.0034	3.51E-41	0.5844	198.4783737
HDL-C	rs4917014	G	T	0.0222	0.0036	1.03E-08	0.3404	38.02777778
HDL-C	rs492571	T	C	0.0663	0.009	1.27E-12	0.95778	54.26777778

HDL-C	rs4939883	C	T	0.0799	0.0045	1.80E-66	0.8193	315.2597531
HDL-C	rs4969178	G	A	0.0263	0.0035	1.53E-12	0.6266	56.4644898
HDL-C	rs4983559	G	A	0.0197	0.0036	9.57E-09	0.3773	29.94521605
HDL-C	rs499974	C	A	0.0263	0.0044	1.12E-08	0.8245	35.72778926
HDL-C	rs6031587	C	T	0.0488	0.0074	1.92E-09	0.9314	43.48867787
HDL-C	rs633695	G	A	0.0885	0.0054	7.82E-58	0.285	268.595679
HDL-C	rs6450176	G	A	0.0254	0.0039	6.88E-10	0.7216	42.41683103
HDL-C	rs6567160	T	C	0.0257	0.0041	2.92E-09	0.7691	39.29149316
HDL-C	rs676210	A	G	0.066	0.004	2.35E-54	0.2309	272.25
HDL-C	rs6805251	T	C	0.02	0.0035	1.33E-08	0.3813	32.65306122
HDL-C	rs686030	A	C	0.055	0.0049	4.29E-27	0.8588	125.9891712
HDL-C	rs687339	C	T	0.0316	0.0042	7.11E-13	0.2335	56.60770975
HDL-C	rs702485	G	A	0.0243	0.0034	6.45E-12	0.4499	51.08044983
HDL-C	rs7112577	G	C	0.0826	0.0129	2.34E-10	0.0399	40.99969954
HDL-C	rs7306660	G	A	0.0345	0.0036	3.34E-19	0.6306	91.84027778
HDL-C	rs731839	A	G	0.022	0.0037	3.44E-09	0.6583	35.35427319
HDL-C	rs737337	T	C	0.0565	0.0061	4.56E-17	0.9314	85.79011019
HDL-C	rs7607980	C	T	0.0447	0.0052	1.81E-15	0.1491	73.89386095
HDL-C	rs838876	A	G	0.0493	0.0039	7.33E-33	0.3259	159.7955293
HDL-C	rs9457931	A	G	0.0552	0.0073	7.30E-13	0.9314	57.1784575
HDL-C	rs964184	C	G	0.1065	0.0071	6.09E-48	0.8616	225
HDL-C	rs970548	C	A	0.0258	0.0039	1.71E-10	0.277	43.76331361

HDL-C	rs998584	C	A	0.026	0.0038	2.27E-11	0.4855	46.81440443
HDL-C	rs9989419	G	A	0.1473	0.0036	########	0.595	1674.173611
LDL-C	rs10195252	T	C	0.0238	0.0039	3.81E-08	0.5818	37.24128863
LDL-C	rs10490626	G	A	0.0508	0.0069	1.70E-12	0.92084	54.20373871
LDL-C	rs10832962	T	C	0.032	0.004	6.62E-14	0.719	64
LDL-C	rs10893499	A	G	0.0521	0.0053	3.86E-21	0.1438	96.63260947
LDL-C	rs10903129	G	A	0.0328	0.0037	3.03E-17	0.5369	78.58582907
LDL-C	rs10947332	A	G	0.0504	0.0056	6.97E-18	0.1319	81
LDL-C	rs112201728	T	C	0.0675	0.0104	8.51E-10	0.05805	42.12509246
LDL-C	rs11563251	T	C	0.0345	0.0062	4.50E-08	0.1253	30.96383975
LDL-C	rs11591147	G	T	0.497	0.018	########	0.98285	762.3734568
LDL-C	rs1169288	C	A	0.0375	0.004	6.45E-21	0.3338	87.890625
LDL-C	rs1250229	C	T	0.0243	0.0042	3.13E-08	0.7889	33.4744898
LDL-C	rs12721109	G	A	0.4462	0.0183	########	0.98285	594.5069724
LDL-C	rs12748152	T	C	0.0499	0.0066	3.21E-12	0.07124	57.162764
LDL-C	rs12916	C	T	0.0733	0.0038	7.79E-78	0.4314	372.083795
LDL-C	rs13206249	G	A	0.0378	0.0062	4.53E-08	0.7836	37.17065557
LDL-C	rs13277801	C	T	0.0338	0.0038	3.99E-17	0.347	79.11634349
LDL-C	rs1367117	A	G	0.1186	0.004	########	0.2876	879.1225
LDL-C	rs1408272	T	G	0.052	0.0083	3.68E-09	0.94723	39.25097982
LDL-C	rs1564348	C	T	0.0481	0.005	2.76E-21	0.1451	92.5444
LDL-C	rs16831243	T	C	0.0378	0.0055	9.06E-12	0.1807	47.23438017

LDL-C	rs16891156	C	A	0.0965	0.0171	8.23E-09	0.01847	31.84655108
LDL-C	rs17404153	G	T	0.0336	0.0054	1.83E-09	0.8562	38.71604938
LDL-C	rs174583	C	T	0.0522	0.0038	7.00E-41	0.6253	188.700831
LDL-C	rs1800961	C	T	0.0685	0.0106	6.03E-10	0.9657	41.76085796
LDL-C	rs1801689	C	A	0.1028	0.0139	9.81E-12	0.03694	54.69613374
LDL-C	rs1883025	C	T	0.0296	0.0044	6.14E-11	0.7573	45.25619835
LDL-C	rs2000999	A	G	0.065	0.0046	4.22E-41	0.1847	199.6691871
LDL-C	rs2030746	T	C	0.0214	0.0038	8.61E-09	0.3984	31.71468144
LDL-C	rs2073547	G	A	0.0485	0.0049	1.92E-21	0.1939	97.969596
LDL-C	rs2228603	C	T	0.104	0.0072	4.43E-44	0.92876	208.6419753
LDL-C	rs2315065	A	C	0.1102	0.0158	5.23E-12	0.08707	48.64621054
LDL-C	rs2328223	C	A	0.0299	0.005	5.63E-09	0.2493	35.7604
LDL-C	rs2390536	A	G	0.0223	0.0038	2.04E-08	0.3681	34.43836565
LDL-C	rs2419604	A	G	0.0302	0.004	7.49E-14	0.3179	57.0025
LDL-C	rs247616	C	T	0.0547	0.0041	2.57E-37	0.7071	177.994646
LDL-C	rs2587534	A	G	0.0391	0.0037	8.06E-25	0.5277	111.6734843
LDL-C	rs2642438	G	A	0.0352	0.0042	7.32E-16	0.7454	70.24036281
LDL-C	rs267733	A	G	0.0331	0.0053	5.29E-09	0.8628	39.00355999
LDL-C	rs2710642	A	G	0.0239	0.0038	6.09E-09	0.6187	39.55747922
LDL-C	rs2737252	G	A	0.0314	0.0041	7.04E-14	0.7441	58.65318263
LDL-C	rs2886232	T	C	0.0451	0.0064	3.88E-11	0.1201	49.65844727
LDL-C	rs2902875	T	C	0.0756	0.0126	2.19E-09	0.0409	36

LDL-C	rs2954029	A	T	0.0564	0.0036	2.10E-50	0.5317	245.4444444
LDL-C	rs2965157	T	C	0.1886	0.0112	7.29E-62	0.97889	283.5615434
LDL-C	rs314253	T	C	0.0242	0.0038	3.44E-10	0.6649	40.5567867
LDL-C	rs3184504	C	T	0.0268	0.0038	4.20E-12	0.5343	49.73961219
LDL-C	rs364585	G	A	0.0249	0.0038	4.28E-10	0.6332	42.93698061
LDL-C	rs3757354	C	T	0.0382	0.0044	2.09E-17	0.7902	75.37396694
LDL-C	rs3780181	A	G	0.0445	0.0074	1.76E-09	0.94723	36.16234478
LDL-C	rs413380	C	T	0.0861	0.0098	7.62E-17	0.9657	77.18877551
LDL-C	rs4253776	G	A	0.0311	0.0059	3.35E-08	0.124	27.78540649
LDL-C	rs4530754	A	G	0.0275	0.0036	3.58E-12	0.5818	58.35262346
LDL-C	rs4722551	C	T	0.0391	0.0049	3.95E-14	0.1702	63.67388588
LDL-C	rs4942486	T	C	0.0243	0.0037	2.26E-11	0.4617	43.13294375
LDL-C	rs4970712	C	A	0.0339	0.0044	2.46E-13	0.8061	59.36002066
LDL-C	rs4970834	C	T	0.1503	0.0047	########	0.8127	1022.638751
LDL-C	rs5763662	T	C	0.0767	0.0121	1.19E-08	0.02507	40.18093026
LDL-C	rs579459	C	T	0.0665	0.0045	2.42E-44	0.215	218.382716
LDL-C	rs6016373	A	G	0.0349	0.0037	7.95E-19	0.6266	88.97078159
LDL-C	rs6065311	C	T	0.0417	0.0036	1.66E-30	0.4604	134.1736111
LDL-C	rs6504872	T	C	0.0274	0.0037	3.48E-13	0.4723	54.84002922
LDL-C	rs6511720	G	T	0.2209	0.0061	########	0.90237	1311.38968
LDL-C	rs6544713	T	C	0.0806	0.0041	4.84E-83	0.2942	386.4580607
LDL-C	rs6709904	A	G	0.055	0.0085	4.58E-10	0.8865	41.86851211

LDL-C	rs676388	C	T	0.0265	0.0039	1.31E-11	0.4631	46.17028271
LDL-C	rs6818397	T	G	0.0224	0.004	1.68E-08	0.4129	31.36
LDL-C	rs6882076	C	T	0.0456	0.0038	3.31E-31	0.6662	144
LDL-C	rs6909746	C	T	0.0263	0.0037	7.86E-11	0.6082	50.52520088
LDL-C	rs7254892	G	A	0.4853	0.0119	########	0.96834	1663.13177
LDL-C	rs72902576	T	G	0.0933	0.0133	9.58E-12	0.96306	49.21075245
LDL-C	rs7534572	G	C	0.0407	0.0058	1.29E-11	0.69	49.24167658
LDL-C	rs7551981	T	G	0.0472	0.0038	1.36E-33	0.595	154.2825485
LDL-C	rs75687619	T	G	0.1735	0.0161	8.05E-24	0.02375	116.1307434
LDL-C	rs7640978	C	T	0.0392	0.0069	9.84E-09	0.8945	32.27557236
LDL-C	rs7832643	T	G	0.0339	0.0038	2.67E-17	0.405	79.58518006
LDL-C	rs8017377	A	G	0.0303	0.0038	2.52E-15	0.4591	63.57963989
LDL-C	rs964184	G	C	0.0855	0.0078	2.01E-26	0.13836	120.1553254
LDL-C	rs9875338	G	A	0.027	0.0037	2.21E-11	0.6121	53.25054785
LDL-C	rs9987289	G	A	0.0714	0.0066	8.53E-24	0.9248	117.0330579
TC	rs10088180	A	G	0.0228	0.004	6.02E-10	0.3219	32.49
TC	rs10468017	T	C	0.0617	0.004	7.23E-48	0.2757	237.930625
TC	rs10773003	A	G	0.0369	0.0058	4.08E-09	0.08839	40.47592152
TC	rs10832962	T	C	0.0315	0.0039	1.54E-14	0.719	65.23668639
TC	rs10900221	A	G	0.0255	0.0041	7.96E-09	0.2731	38.68233195
TC	rs10904908	G	A	0.025	0.0036	2.60E-11	0.4538	48.22530864
TC	rs11153594	C	T	0.029	0.0036	1.27E-14	0.6082	64.89197531

TC	rs112201728	T	C	0.0581	0.0099	1.20E-08	0.05805	34.44148556
TC	rs11220462	A	G	0.0474	0.0058	5.49E-15	0.1425	66.78834721
TC	rs11563251	T	C	0.0368	0.0059	1.27E-09	0.1253	38.90376329
TC	rs11591147	G	T	0.3341	0.0173	8.83E-86	0.98285	372.9587023
TC	rs11694172	G	A	0.0277	0.0041	1.95E-09	0.2164	45.64485425
TC	rs11753995	A	G	0.0489	0.0048	1.84E-23	0.1464	103.7851563
TC	rs11789603	T	C	0.0427	0.0062	1.44E-11	0.08971	47.43210198
TC	rs11802413	T	C	0.0287	0.0035	1.58E-14	0.5369	67.24
TC	rs12412743	C	T	0.0298	0.0047	6.98E-10	0.847	40.20099593
TC	rs12670798	C	T	0.0364	0.0041	9.48E-17	0.2243	78.81975015
TC	rs12916	C	T	0.0684	0.0036	4.55E-74	0.4314	361
TC	rs13315871	G	A	0.0355	0.0061	3.48E-08	0.91953	33.86858371
TC	rs138777	A	G	0.0214	0.0037	4.74E-08	0.3483	33.45215486
TC	rs1535	A	G	0.0497	0.0037	8.62E-39	0.6372	180.4302411
TC	rs17526895	A	G	0.042	0.0067	5.78E-09	0.92216	39.29605703
TC	rs1800562	G	A	0.0565	0.0077	1.91E-12	0.95383	53.84128858
TC	rs1800961	C	T	0.1062	0.0101	1.34E-24	0.9657	110.5621018
TC	rs181360	T	G	0.0278	0.0043	7.32E-10	0.8008	41.7977285
TC	rs1883025	C	T	0.0671	0.0042	5.75E-53	0.7573	255.2386621
TC	rs1997243	G	A	0.0332	0.005	2.72E-10	0.1306	44.0896
TC	rs2000999	A	G	0.0617	0.0044	6.80E-41	0.1847	196.6368802
TC	rs2030746	T	C	0.0199	0.0037	3.60E-08	0.3984	28.92695398

TC	rs2066714	C	T	0.0442	0.0076	1.14E-08	0.1201	33.8234072
TC	rs2073547	G	A	0.0456	0.0047	3.83E-21	0.1939	94.13128112
TC	rs2156552	T	A	0.057	0.0047	1.25E-31	0.8219	147.0801268
TC	rs2228603	C	T	0.1217	0.0069	1.05E-62	0.92876	311.0877967
TC	rs2235367	G	A	0.0357	0.0035	7.22E-25	0.4565	104.04
TC	rs2244608	G	A	0.0313	0.0037	9.62E-18	0.3391	71.56245435
TC	rs2255141	A	G	0.0314	0.0039	6.51E-16	0.3193	64.82314267
TC	rs2277862	C	T	0.0349	0.0052	5.26E-11	0.8681	45.04474852
TC	rs2287623	G	A	0.0273	0.0036	4.09E-12	0.405	57.50694444
TC	rs2315065	A	C	0.1102	0.0158	1.10E-11	0.08707	48.64621054
TC	rs247616	T	C	0.0499	0.004	4.47E-32	0.2929	155.625625
TC	rs2642438	G	A	0.037	0.004	1.28E-18	0.7454	85.5625
TC	rs2737252	G	A	0.0331	0.0039	1.63E-16	0.7441	72.03221565
TC	rs281393	C	T	0.0322	0.0055	4.26E-08	0.6266	34.27570248
TC	rs2814982	C	T	0.0441	0.0057	3.68E-15	0.8931	59.85872576
TC	rs2886232	T	C	0.0358	0.0062	3.87E-08	0.1201	33.34131113
TC	rs2902875	T	C	0.0707	0.0123	1.79E-08	0.0409	33.03913015
TC	rs2954029	A	T	0.0622	0.0035	2.42E-65	0.5317	315.8236735
TC	rs314253	T	C	0.0233	0.0037	2.81E-10	0.6649	39.65595325
TC	rs3184504	C	T	0.0318	0.0037	1.62E-17	0.5343	73.86705625
TC	rs3757354	C	T	0.0348	0.0042	2.22E-15	0.7902	68.65306122
TC	rs3780181	A	G	0.0442	0.0071	6.67E-10	0.94723	38.75500893

TC	rs4253772	T	C	0.0322	0.0058	9.85E-09	0.1187	30.8216409
TC	rs4530754	A	G	0.0228	0.0035	1.68E-09	0.5818	42.43591837
TC	rs4738684	A	G	0.0392	0.0037	1.12E-23	0.3522	112.2454346
TC	rs4752805	G	A	0.0251	0.0041	1.62E-09	0.2467	37.47828673
TC	rs4988235	G	A	0.0308	0.004	3.98E-14	0.4763	59.29
TC	rs515135	C	T	0.1238	0.0046	########	0.7823	724.3119093
TC	rs558971	G	A	0.0398	0.0036	7.03E-28	0.5303	122.2253086
TC	rs579459	C	T	0.062	0.0044	8.83E-42	0.215	198.553719
TC	rs581080	C	G	0.0377	0.0047	1.02E-13	0.8206	64.34087823
TC	rs6016373	A	G	0.0319	0.0036	1.00E-17	0.6266	78.51929012
TC	rs633695	G	A	0.0433	0.0058	1.05E-14	0.285	55.73394768
TC	rs646776	T	C	0.1272	0.0042	########	0.7876	917.2244898
TC	rs6504872	T	C	0.025	0.0035	6.99E-12	0.4723	51.02040816
TC	rs6511720	G	T	0.1851	0.0059	########	0.90237	984.2576846
TC	rs6544713	T	C	0.0773	0.004	1.69E-81	0.2942	373.455625
TC	rs6573778	T	C	0.0263	0.0039	2.96E-11	0.471	45.47600263
TC	rs6603981	T	C	0.0351	0.0043	7.85E-15	0.8061	66.63115197
TC	rs6709904	A	G	0.0545	0.0083	8.40E-10	0.8865	43.11583684
TC	rs6818397	T	G	0.0254	0.0039	9.51E-11	0.4129	42.41683103
TC	rs6882076	C	T	0.0508	0.0037	5.35E-41	0.6662	188.5054785
TC	rs7412	C	T	0.3736	0.0096	########	0.93404	1514.506944
TC	rs7534572	G	C	0.0629	0.0055	3.60E-28	0.69	130.7904132

TC	rs7551981	T	G	0.0358	0.0037	7.50E-22	0.595	93.61869978
TC	rs75687619	T	G	0.1592	0.0153	3.61E-22	0.02375	108.2687855
TC	rs7616006	A	G	0.0315	0.0036	8.41E-17	0.5554	76.5625
TC	rs7640978	C	T	0.0376	0.0066	1.66E-08	0.8945	32.45546373
TC	rs780093	T	C	0.0515	0.0036	2.59E-42	0.4129	204.6489198
TC	rs7832643	T	G	0.0289	0.0037	3.12E-13	0.405	61.00876552
TC	rs8103315	A	C	0.0422	0.0055	5.94E-15	0.1359	58.8707438
TC	rs9272775	C	T	0.0317	0.0055	2.13E-08	0.2823	33.21950413
TC	rs9306897	T	C	0.0488	0.0037	7.52E-37	0.3034	173.9547115
TC	rs9376090	T	C	0.0254	0.004	2.60E-09	0.7282	40.3225
TC	rs9391858	G	A	0.0495	0.005	7.20E-22	0.1939	98.01
TC	rs964184	G	C	0.1214	0.0076	2.84E-55	0.13836	255.1585873
TC	rs9987289	G	A	0.0842	0.0063	1.84E-36	0.9248	178.6253464
TG	rs10401969	T	C	0.121	0.0065	9.70E-70	0.92876	346.5325444
TG	rs10440120	C	A	0.0306	0.0044	5.34E-11	0.8325	48.36570248
TG	rs10501321	T	C	0.0216	0.0035	1.41E-08	0.686	38.08653061
TG	rs10761762	T	C	0.027	0.0033	1.06E-17	0.533	66.94214876
TG	rs11057408	G	T	0.0258	0.0035	2.05E-12	0.6372	54.33795918
TG	rs11613352	C	T	0.028	0.0039	9.40E-14	0.8087	51.54503616
TG	rs11974409	A	G	0.0899	0.0042	########	0.8061	458.1638322
TG	rs12280753	T	C	0.1931	0.0064	########	0.06728	910.342041
TG	rs1260326	T	C	0.1148	0.0034	########	0.4129	1140.055363

TG	rs12676857	C	T	0.0332	0.0046	7.29E-12	0.1544	52.09073724
TG	rs12678919	A	G	0.1702	0.0056	########	0.8786	923.7257653
TG	rs12748152	T	C	0.0372	0.0059	1.10E-09	0.07124	39.75409365
TG	rs1321257	G	A	0.0402	0.0034	5.99E-31	0.4063	139.7958478
TG	rs13389219	C	T	0.0271	0.0034	2.60E-15	0.591	63.53027682
TG	rs16948098	A	G	0.08	0.0089	4.84E-17	0.0409	80.79787906
TG	rs174535	C	T	0.047	0.0034	1.73E-41	0.3628	191.0899654
TG	rs17513135	T	C	0.022	0.0039	1.63E-08	0.2322	31.82117028
TG	rs1800775	C	A	0.0396	0.0035	1.33E-26	0.5198	128.0130612
TG	rs1832007	A	G	0.0327	0.0047	1.72E-12	0.8681	48.40606609
TG	rs2043085	T	C	0.0327	0.0034	7.81E-20	0.3681	92.49913495
TG	rs2068888	G	A	0.0241	0.0034	1.68E-11	0.5092	50.24307958
TG	rs2239520	G	A	0.0236	0.0037	4.14E-10	0.6266	40.68371074
TG	rs2247056	C	T	0.0378	0.0039	3.86E-21	0.7823	93.9408284
TG	rs2250802	A	G	0.023	0.0037	1.21E-10	0.6807	38.64134405
TG	rs2665357	C	A	0.0212	0.0033	8.33E-10	0.5092	41.27089073
TG	rs287621	T	C	0.0222	0.0037	7.67E-09	0.2704	36
TG	rs2954022	C	A	0.078	0.0033	########	0.5303	558.677686
TG	rs2972146	T	G	0.0281	0.0034	2.97E-15	0.6227	68.30536332
TG	rs3198697	C	T	0.0198	0.0034	2.21E-08	0.6174	33.91349481
TG	rs3760627	C	T	0.0189	0.0034	5.29E-09	0.4683	30.90051903
TG	rs3761445	A	G	0.0232	0.0034	8.06E-12	0.6148	46.56055363

TG	rs38855	A	G	0.0187	0.0033	2.11E-08	0.5264	32.11111111
TG	rs439401	C	T	0.0659	0.0038	1.42E-66	0.6201	300.748615
TG	rs442177	T	G	0.0309	0.0033	1.32E-18	0.5528	87.67768595
TG	rs4587594	G	A	0.0694	0.0035	3.50E-82	0.69	393.1722449
TG	rs4719841	G	A	0.0232	0.0034	8.86E-11	0.3826	46.56055363
TG	rs4738684	A	G	0.0205	0.0035	8.82E-09	0.3522	34.30612245
TG	rs4810479	C	T	0.0474	0.0038	2.07E-34	0.2876	155.5927978
TG	rs588136	C	T	0.0495	0.0041	3.37E-30	0.2058	145.7614515
TG	rs6029143	C	T	0.0388	0.0071	4.93E-08	0.94195	29.86391589
TG	rs634869	T	C	0.0272	0.0033	1.78E-14	0.438	67.93755739
TG	rs645040	T	G	0.0293	0.004	1.83E-12	0.7691	53.655625
TG	rs676210	G	A	0.0733	0.0039	3.28E-71	0.7691	353.2472058
TG	rs6831256	G	A	0.0258	0.0035	1.60E-12	0.409	54.33795918
TG	rs6882076	C	T	0.0286	0.0035	1.51E-15	0.6662	66.7722449
TG	rs6995541	G	A	0.0265	0.0037	1.34E-12	0.3219	51.29656684
TG	rs719726	T	C	0.0199	0.0035	2.49E-08	0.529	32.32734694
TG	rs7248104	G	A	0.0222	0.0034	5.05E-10	0.5831	42.63321799
TG	rs731839	G	A	0.0224	0.0036	2.65E-09	0.3417	38.71604938
TG	rs7350481	T	C	0.2254	0.0066	########	0.09763	1166.325987
TG	rs749671	G	A	0.0211	0.0034	6.11E-10	0.6055	38.51297578
TG	rs8077889	C	A	0.0252	0.0042	9.88E-09	0.2441	36
TG	rs948690	T	C	0.0306	0.0052	6.57E-09	0.6953	34.62869822

TG	rs9686661	T	C	0.0379	0.0044	2.54E-16	0.1768	74.1947314
TG	rs998584	A	C	0.0293	0.0037	3.42E-15	0.5145	62.70927684

Table S3. All Mendelian randomization analysis results of lipid traits.

Exposure	Outcome	Cohort	Method	Snp (N)	Beta	Se	Pvalue	MR-Egger intercept	MR-Egger SE	MR-Egger P-value
HDL-C	cerebral aneurysm	Mark K. Bakker group	MR Egger	71	-0.02	0.15	9.07E-01	-0.005808479	0.006937	0.405298
HDL-C	cerebral aneurysm	Mark K. Bakker group	Inverse variance weighted	71	-0.12	0.08	1.44E-01			
HDL-C	cerebral aneurysm	Mark K. Bakker group	Weighted median	71	-0.05	0.09	5.77E-01			
HDL-C	cerebral aneurysm	Mark K. Bakker group	Weighted mode	71	-0.09	0.09	3.50E-01			
LDL-C	cerebral aneurysm	Mark K. Bakker group	MR Egger	60	-0.11	0.10	2.89E-01	0.003473388	0.006082	0.570127
LDL-C	cerebral aneurysm	Mark K. Bakker group	Weighted median	60	0.00	0.08	9.58E-01			
LDL-C	cerebral aneurysm	Mark K. Bakker group	Weighted mode	60	-0.05	0.08	4.83E-01			
LDL-C	cerebral aneurysm	Mark K. Bakker group	Inverse variance weighted	60	-0.06	0.07	3.24E-01			

TC	cerebral aneurysm	Mark K. Bakker group	MR Egger	64	-0.11	0.09	2.25E-01	0.004475566	0.005333	0.404581
TC	cerebral aneurysm	Mark K. Bakker group	Weighted median	64	-0.04	0.09	6.38E-01			
TC	cerebral aneurysm	Mark K. Bakker group	Weighted mode	64	-0.05	0.07	4.61E-01			
TC	cerebral aneurysm	Mark K. Bakker group	Inverse variance weighted	64	-0.05	0.06	3.63E-01			
TG	cerebral aneurysm	Mark K. Bakker group	MR Egger	47	0.03	0.13	8.37E-01	0.009770639	0.006702	0.151824
TG	cerebral aneurysm	Mark K. Bakker group	Weighted median	47	0.13	0.11	2.41E-01			
TG	cerebral aneurysm	Mark K. Bakker group	Weighted mode	47	0.15	0.10	1.47E-01			
TG	cerebral aneurysm	Mark K. Bakker group	Inverse variance weighted	47	0.17	0.08	4.45E-02			
HDL-C	cerebral aneurysm	UK Biobank	Inverse variance weighted	88	-0.29	0.21	1.62E-01	0.002828906	0.017692	0.873336
HDL-C	cerebral aneurysm	UK Biobank	MR Egger	88	-0.34	0.39	3.80E-01			

HDL-C	cerebral aneurysm	UK Biobank	Weighted median	88	-0.51	0.29	8.18E-02			
HDL-C	cerebral aneurysm	UK Biobank	Weighted mode	88	-0.43	0.27	1.16E-01			
LDL-C	cerebral aneurysm	UK Biobank	Inverse variance weighted	79	-0.21	0.15	1.75E-01	0.003091762	0.014663	0.833558
LDL-C	cerebral aneurysm	UK Biobank	MR Egger	79	-0.24	0.22	2.85E-01			
LDL-C	cerebral aneurysm	UK Biobank	Weighted median	79	0.03	0.23	8.78E-01			
LDL-C	cerebral aneurysm	UK Biobank	Weighted mode	79	-0.06	0.22	7.77E-01			
TC	cerebral aneurysm	UK Biobank	Inverse variance weighted	86	-0.20	0.15	1.68E-01	0.012590262	0.013857	0.366164
TC	cerebral aneurysm	UK Biobank	MR Egger	86	-0.37	0.24	1.21E-01			
TC	cerebral aneurysm	UK Biobank	Weighted median	86	-0.29	0.25	2.45E-01			

TC	cerebral aneurysm	UK Biobank	Weighted mode	86	-0.25	0.23	2.82E-01			
TG	cerebral aneurysm	UK Biobank	MR Egger	55	-0.03	0.33	9.18E-01	0.029076338	0.015884	0.072792
TG	cerebral aneurysm	UK Biobank	Weighted median	55	0.08	0.30	8.00E-01			
TG	cerebral aneurysm	UK Biobank	Weighted mode	55	0.16	0.28	5.61E-01			
TG	cerebral aneurysm	UK Biobank	Inverse variance weighted	55	0.43	0.20	3.22E-02			
HDL-C	Aortic aneurysm	UK Biobank	MR Egger	88	-0.24	0.19	2.18E-01	-0.007170789	0.008828	0.418891
HDL-C	Aortic aneurysm	UK Biobank	Weighted median	88	-0.51	0.15	4.82E-04			
HDL-C	Aortic aneurysm	UK Biobank	Weighted mode	88	-0.44	0.16	7.00E-03			
HDL-C	Aortic aneurysm	UK Biobank	Inverse variance weighted	88	-0.37	0.10	3.30E-04			
LDL-C	Aortic aneurysm	UK Biobank	MR Egger	79	0.28	0.12	2.13E-02	0.009540292	0.007914	0.231689

LDL-C	Aortic aneurysm	UK Biobank	Inverse variance weighted	79	0.39	0.08	2.54E-06			
LDL-C	Aortic aneurysm	UK Biobank	Weighted median	79	0.32	0.12	1.09E-02			
LDL-C	Aortic aneurysm	UK Biobank	Weighted mode	79	0.27	0.11	1.47E-02			
TC	Aortic aneurysm	UK Biobank	Inverse variance weighted	86	0.34	0.08	7.12E-05	-0.005651234	0.007998	0.481756
TC	Aortic aneurysm	UK Biobank	MR Egger	86	0.41	0.14	3.50E-03			
TC	Aortic aneurysm	UK Biobank	Weighted median	86	0.21	0.12	8.39E-02			
TC	Aortic aneurysm	UK Biobank	Weighted mode	86	0.28	0.13	3.12E-02			
TG	Aortic aneurysm	UK Biobank	Inverse variance weighted	55	0.30	0.11	8.18E-03	-0.004797518	0.009051	0.598281
TG	Aortic aneurysm	UK Biobank	MR Egger	55	0.38	0.19	4.55E-02			
TG	Aortic aneurysm	UK Biobank	Weighted median	55	0.28	0.15	5.80E-02			
TG	Aortic aneurysm	UK Biobank	Weighted mode	55	0.33	0.14	2.79E-02			
HDL-C	Aortic aneurysm	FinnGen	MR Egger	84	-0.07	0.18	6.93E-01	-0.009428291	0.008045	0.244596

HDL-C	Aortic aneurysm	FinnGen	Weighted median	84	-0.16	0.13	1.99E-01			
HDL-C	Aortic aneurysm	FinnGen	Weighted mode	84	-0.13	0.13	2.99E-01			
HDL-C	Aortic aneurysm	FinnGen	Inverse variance weighted	84	-0.24	0.09	9.83E-03			
LDL-C	Aortic aneurysm	FinnGen	MR Egger	75	0.27	0.12	2.47E-02	0.003071608	0.007595	0.687101
LDL-C	Aortic aneurysm	FinnGen	Weighted median	75	0.21	0.10	3.54E-02			
LDL-C	Aortic aneurysm	FinnGen	Weighted mode	75	0.22	0.09	1.84E-02			
LDL-C	Aortic aneurysm	FinnGen	Inverse variance weighted	75	0.30	0.08	2.15E-04			
TC	Aortic aneurysm	FinnGen	Inverse variance weighted	76	0.27	0.09	2.55E-03	0.002924086	0.008275	0.724811
TC	Aortic aneurysm	FinnGen	MR Egger	76	0.23	0.15	1.10E-01			
TC	Aortic aneurysm	FinnGen	Weighted median	76	0.35	0.12	4.03E-03			
TC	Aortic aneurysm	FinnGen	Weighted mode	76	0.26	0.11	2.65E-02			

TG	Aortic aneurysm	FinnGen	Inverse variance weighted	54	0.31	0.09	1.17E-03	0.007286607	0.007448	0.332447
TG	Aortic aneurysm	FinnGen	MR Egger	54	0.19	0.15	2.07E-01			
TG	Aortic aneurysm	FinnGen	Weighted median	54	0.12	0.14	4.00E-01			
TG	Aortic aneurysm	FinnGen	Weighted mode	54	0.13	0.13	3.52E-01			
HDL-C	Abdominal aortic aneurysm	UK Biobank	MR Egger	87	-0.46	0.24	6.59E-02	-0.004975233	0.011248	0.659375
HDL-C	Abdominal aortic aneurysm	UK Biobank	Inverse variance weighted	87	-0.55	0.13	3.09E-05			
HDL-C	Abdominal aortic aneurysm	UK Biobank	Weighted median	87	-0.46	0.18	9.00E-03			
HDL-C	Abdominal aortic aneurysm	UK Biobank	Weighted mode	87	-0.48	0.19	1.10E-02			
LDL-C	Abdominal aortic aneurysm	UK Biobank	MR Egger	79	0.54	0.15	5.88E-04	0.004661398	0.009919	0.639738
LDL-C	Abdominal aortic aneurysm	UK Biobank	Inverse variance weighted	79	0.60	0.10	8.00E-09			
LDL-C	Abdominal aortic aneurysm	UK Biobank	Weighted median	79	0.42	0.16	7.79E-03			

LDL-C	Abdominal aortic aneurysm	UK Biobank	Weighted mode	79	0.38	0.16	1.99E-02			
TC	Abdominal aortic aneurysm	UK Biobank	MR Egger	85	0.65	0.18	5.28E-04	-0.014790597	0.010182	0.150115
TC	Abdominal aortic aneurysm	UK Biobank	Inverse variance weighted	85	0.44	0.11	7.22E-05			
TC	Abdominal aortic aneurysm	UK Biobank	Weighted median	85	0.34	0.16	3.37E-02			
TC	Abdominal aortic aneurysm	UK Biobank	Weighted mode	85	0.44	0.17	1.25E-02			
TG	Abdominal aortic aneurysm	UK Biobank	MR Egger	55	0.32	0.23	1.67E-01	0.004880863	0.011228	0.665538
TG	Abdominal aortic aneurysm	UK Biobank	Inverse variance weighted	55	0.40	0.14	4.78E-03			
TG	Abdominal aortic aneurysm	UK Biobank	Weighted median	55	0.38	0.19	5.01E-02			
TG	Abdominal aortic aneurysm	UK Biobank	Weighted mode	55	0.47	0.19	1.65E-02			
HDL-C	Iliac artery aneurysm	UK Biobank	MR Egger	88	0.15	0.65	8.17E-01	-0.01321541	0.030038	0.661076
HDL-C	Iliac artery aneurysm	UK Biobank	Inverse variance weighted	88	-0.09	0.35	7.96E-01			
HDL-C	Iliac artery aneurysm	UK Biobank	Weighted median	88	-0.51	0.54	3.50E-01			

HDL-C	Iliac artery aneurysm	UK Biobank	Weighted mode	88	-0.70	0.55	2.05E-01			
LDL-C	Iliac artery aneurysm	UK Biobank	MR Egger	78	0.37	0.46	4.24E-01	-0.007307121	0.028824	0.800558
LDL-C	Iliac artery aneurysm	UK Biobank	Inverse variance weighted	78	0.28	0.30	3.50E-01			
LDL-C	Iliac artery aneurysm	UK Biobank	Weighted median	78	-0.14	0.44	7.59E-01			
LDL-C	Iliac artery aneurysm	UK Biobank	Weighted mode	78	0.24	0.43	5.78E-01			
TC	Iliac artery aneurysm	UK Biobank	MR Egger	85	0.19	0.49	7.02E-01	-0.012380782	0.028237	0.662195
TC	Iliac artery aneurysm	UK Biobank	Inverse variance weighted	85	0.02	0.30	9.53E-01			
TC	Iliac artery aneurysm	UK Biobank	Weighted median	85	0.09	0.49	8.56E-01			
TC	Iliac artery aneurysm	UK Biobank	Weighted mode	85	0.02	0.49	9.65E-01			
TG	Iliac artery aneurysm	UK Biobank	MR Egger	55	0.82	0.61	1.86E-01	-0.05495947	0.029827	0.070984
TG	Iliac artery aneurysm	UK Biobank	Inverse variance weighted	55	-0.06	0.39	8.71E-01			

TG	Iliac artery aneurysm	UK Biobank	Weighted median	55	0.29	0.55	5.98E-01			
TG	Iliac artery aneurysm	UK Biobank	Weighted mode	55	0.53	0.47	2.68E-01			
HDL-C	Lower extremity artery aneurysm	UK Biobank	MR Egger	88	-0.25	0.49	6.13E-01	0.018523993	0.022497	0.412558
HDL-C	Lower extremity artery aneurysm	UK Biobank	Inverse variance weighted	88	0.09	0.26	7.28E-01			
HDL-C	Lower extremity artery aneurysm	UK Biobank	Weighted median	88	0.01	0.43	9.74E-01			
HDL-C	Lower extremity artery aneurysm	UK Biobank	Weighted mode	88	0.08	0.46	8.70E-01			
LDL-C	Lower extremity artery aneurysm	UK Biobank	MR Egger	79	0.00	0.33	9.94E-01	0.021293356	0.021299	0.32058
LDL-C	Lower extremity artery aneurysm	UK Biobank	Inverse variance weighted	79	0.24	0.22	2.82E-01			
LDL-C	Lower extremity artery aneurysm	UK Biobank	Weighted median	79	0.04	0.34	9.12E-01			
LDL-C	Lower extremity artery aneurysm	UK Biobank	Weighted mode	79	-0.08	0.32	7.91E-01			
TC	Lower extremity artery aneurysm	UK Biobank	MR Egger	86	-0.14	0.37	7.00E-01	0.031359079	0.021754	0.153148

TC	Lower extremity artery aneurysm	UK Biobank	Inverse variance weighted	86	0.28	0.23	2.27E-01			
TC	Lower extremity artery aneurysm	UK Biobank	Weighted median	86	0.18	0.37	6.37E-01			
TC	Lower extremity artery aneurysm	UK Biobank	Weighted mode	86	0.70	0.72	3.36E-01			
TG	Lower extremity artery aneurysm	UK Biobank	MR Egger	55	1.51	0.51	4.35E-03	-0.030890114	0.024789	0.218199
TG	Lower extremity artery aneurysm	UK Biobank	Inverse variance weighted	55	1.02	0.32	1.34E-03			
TG	Lower extremity artery aneurysm	UK Biobank	Weighted median	55	1.25	0.47	7.39E-03			
TG	Lower extremity artery aneurysm	UK Biobank	Weighted mode	55	1.44	0.44	2.03E-03			

Table S4. Multivariable mendelian randomization analysis results of lipid traits with adjustment for HDL-C, LDL-C and TG.

Exposure	Outcome	Cohort	Beta	Se	OR	95% CI	Pvalue
HDL-C	cerebral aneurysm	Mark K. Bakker group	-0.05	0.07	0.95	0.81-1.08	4.47E-01
LDL-C	cerebral aneurysm	Mark K. Bakker group	-0.07	0.07	0.94	0.80-1.07	3.39E-01
TG	cerebral aneurysm	Mark K. Bakker group	0.19	0.10	1.21	1.03-1.40	4.13E-02
HDL-C	cerebral aneurysm	UK Biobank	-0.17	0.17	0.85	0.51-1.18	3.32E-01
LDL-C	cerebral aneurysm	UK Biobank	-0.42	0.15	0.66	0.35-0.96	6.21E-03
TG	cerebral aneurysm	UK Biobank	0.24	0.24	1.27	0.81-1.74	3.09E-01
HDL-C	Aortic aneurysm	UK Biobank	-0.25	0.09	0.78	0.60-0.96	7.85E-03
LDL-C	Aortic aneurysm	UK Biobank	0.10	0.08	1.11	0.94-1.27	2.27E-01
TG	Aortic aneurysm	UK Biobank	0.09	0.13	1.09	0.84-1.34	5.05E-01
HDL-C	Aortic aneurysm	FinnGen	-0.14	0.09	0.87	0.68-1.05	1.27E-01
LDL-C	Aortic aneurysm	FinnGen	0.15	0.07	1.16	1.02-1.31	4.17E-02
TG	Aortic aneurysm	FinnGen	0.12	0.12	1.13	0.89-1.37	3.15E-01
HDL-C	Abdominal aortic aneurysm	UK Biobank	-0.40	0.12	0.67	0.43-0.90	7.13E-04
LDL-C	Abdominal aortic aneurysm	UK Biobank	0.27	0.11	1.31	1.10-1.52	1.23E-02
TG	Abdominal aortic aneurysm	UK Biobank	0.05	0.16	1.05	0.73-1.38	7.47E-01
HDL-C	Iliac artery aneurysm	UK Biobank	0.10	0.32	1.10	0.48-1.73	7.54E-01
LDL-C	Iliac artery aneurysm	UK Biobank	0.13	0.30	1.13	0.55-1.72	6.74E-01
TG	Iliac artery aneurysm	UK Biobank	0.21	0.44	1.23	0.36-2.10	6.38E-01
HDL-C	Lower extremity artery aneurysm	UK Biobank	0.32	0.24	1.38	0.91-1.85	1.81E-01
LDL-C	Lower extremity artery aneurysm	UK Biobank	0.17	0.22	1.19	0.77-1.61	4.22E-01
TG	Lower extremity artery aneurysm	UK Biobank	0.77	0.33	2.16	1.50-2.81	2.08E-02

Table S5. Detailed summary of LDL-C lowering genetic variants in HMGCR, NPC1L1, CETP, PCSK9, LDLR and TG lowering genetic variants in ANGPTL3, LPL.

**Target**	**SNP**	**Pathway**	**Effect_allele**	**Other_allele**	**Beta**	**Se**	**Pvalue**	**Eaf**
HMGCR	rs10515198	LDL-C	G	A	-0.0599	0.0061	5.99E-22	0.1029
HMGCR	rs12916	LDL-C	T	C	-0.0733	0.0038	7.79E-78	0.4314
HMGCR	rs3857388	LDL-C	T	C	-0.0421	0.0059	2.20E-11	0.128
HMGCR	rs4704220	LDL-C	G	A	-0.0639	0.0037	5.13E-62	0.4156
HMGCR	rs9942407	LDL-C	G	A	-0.0489	0.0053	3.13E-19	0.4182
NPC1L1	rs2073547	LDL-C	A	G	-0.0485	0.0049	1.92E-21	0.1939
NPC1L1	rs217386	LDL-C	A	G	-0.0363	0.0038	1.20E-19	0.5923
NPC1L1	rs7791240	LDL-C	T	C	-0.0425	0.0065	1.84E-10	0.09103
PCSK9	rs10493176	LDL-C	G	T	-0.0776	0.0102	2.54E-14	0.8852
PCSK9	rs11206510	LDL-C	C	T	-0.0831	0.005	2.38E-53	0.8456
PCSK9	rs11206514	LDL-C	C	A	-0.0507	0.0041	9.95E-33	0.6108
PCSK9	rs11583974	LDL-C	G	A	-0.0646	0.0117	3.95E-09	0.03034
PCSK9	rs11591147	LDL-C	T	G	-0.497	0.018	8.58E-143	0.98285

PCSK9	rs12067569	LDL-C	G	A	-0.0885	0.01	1.97E-17	0.0343
PCSK9	rs2479394	LDL-C	A	G	-0.0386	0.0041	1.58E-19	0.285
PCSK9	rs2479409	LDL-C	A	G	-0.0642	0.0041	2.52E-50	0.3325
PCSK9	rs2495477	LDL-C	C	T	-0.064	0.0054	7.29E-30	0.596716
PCSK9	rs572512	LDL-C	C	T	-0.0478	0.0047	5.31E-26	0.3456
PCSK9	rs585131	LDL-C	C	T	-0.0637	0.005	2.70E-35	0.8153
CETP	rs12448528	LDL-C	G	A	-0.037	0.0052	1.06E-12	0.2269
CETP	rs1864163	LDL-C	G	A	-0.0437	0.0045	7.97E-21	0.2678
CETP	rs247616	LDL-C	T	C	-0.0547	0.0041	2.57E-37	0.7071
LDLR	rs12052058	LDL-C	T	G	-0.075	0.0043	9.66E-62	0.752
LDLR	rs12983316	LDL-C	A	G	-0.0514	0.0052	7.44E-22	0.1689
LDLR	rs1799898	LDL-C	T	C	-0.0333	0.0054	1.96E-09	0.8496
LDLR	rs2738464	LDL-C	G	C	-0.0422	0.0061	2.73E-10	0.8747
LDLR	rs3786721	LDL-C	C	T	-0.0468	0.0038	2.89E-31	0.4617
LDLR	rs379309	LDL-C	T	C	-0.0313	0.0039	1.39E-13	0.5026

LDLR	rs5742911	LDL-C	G	A	-0.0606	0.0057	4.83E-24	0.7322
LDLR	rs6511720	LDL-C	T	G	-0.2209	0.0061	1.00E-200	0.90237
LDLR	rs688	LDL-C	C	T	-0.054	0.0037	1.01E-43	0.4472
LDLR	rs7251031	LDL-C	T	G	-0.0456	0.0046	6.24E-23	0.2889
LDLR	rs73015030	LDL-C	A	G	-0.1517	0.0148	2.62E-22	0.97493
LDLR	rs892114	LDL-C	G	A	-0.0353	0.0047	7.63E-13	0.2269
ANGPTL3	rs12749263	TG	T	C	-0.0306	0.0039	2.55E-15	0.2573
ANGPTL3	rs13375691	TG	T	C	-0.0485	0.0058	2.54E-16	0.93272
ANGPTL3	rs4587594	TG	A	G	-0.0694	0.0035	3.50E-82	0.69
LPL	rs117604010	TG	A	G	-0.1371	0.017	3.11E-15	0.98021
LPL	rs117910839	TG	A	T	-0.1365	0.0141	7.07E-20	0.96306
LPL	rs12678919	TG	G	A	-0.1702	0.0056	1.82E-199	0.8786
LPL	rs1441771	TG	T	C	-0.0669	0.0043	1.61E-50	0.8364
LPL	rs1534649	TG	T	G	-0.028	0.0035	1.75E-15	0.5515
LPL	rs1899351	TG	G	A	-0.1236	0.0156	2.05E-12	0.01319

LPL	rs283	TG	C	T	-0.037	0.0044	1.70E-16	0.2348
LPL	rs301	TG	C	T	-0.1089	0.0039	1.86E-167	0.5726
LPL	rs4557718	TG	T	C	-0.0578	0.0059	3.26E-24	0.1266
LPL	rs4922116	TG	A	G	-0.0435	0.0044	8.46E-24	0.8522
LPL	rs4922119	TG	T	C	-0.0708	0.0034	1.67E-95	0.5501
LPL	rs6586872	TG	A	G	-0.0339	0.0038	4.09E-19	0.7441
LPL	rs7003579	TG	C	T	-0.0255	0.004	4.72E-10	0.215
LPL	rs7016529	TG	T	C	-0.1911	0.014	3.57E-35	0.01319
LPL	rs894210	TG	A	G	-0.0671	0.0033	2.94E-89	0.4354
